# The past, present and future use of technology-enabled physical activity interventions in clinical and non-clinical populations: a bibliometric trend analysis across four decades

**DOI:** 10.3389/fdgth.2026.1801405

**Published:** 2026-05-29

**Authors:** Hannes Baumann, George Thomas, Stephanie Alley, Mitch J. Duncan, Meighan Browne, Bettina Wollesen, Corneel Vandelanotte, Nicholas Gilson

**Affiliations:** 1 Department of Movement Therapy and Movement-oriented Prevention and Rehabilitation, German Sport University Cologne, Cologne, Germany; 2 Health and Wellbeing Centre for Research Innovation, School of Human Movement and Nutrition Sciences, The University of Queensland, Brisbane, QLD, Australia; 3 Australian Research Council Centre of Excellence for the Digital Child, Brisbane, Australia; 4 School of Health, Medical and Applied Sciences, Appleton Institute, Central Queensland University, Rockhampton, QLD, Australia; 5 School of Medicine and Public Health, University of Newcastle, Newcastle, NSW, Australia; 6 School of Human Movement and Nutrition Sciences, The University of Queensland, Brisbane, QLD, Australia

**Keywords:** artificial intelligence, bibliometric analysis, digital health, mHealth, physical activity, telehealth, virtual reality, wearable sensors

## Abstract

**Background:**

Physical inactivity remains a persistent global health challenge despite long-standing evidence that regular physical activity (PA) reduces chronic disease risk, cognitive decline and premature mortality. In parallel, digital health technologies have expanded rapidly, yet it remains unclear how distinct platform types have emerged, diffused and been differentially adopted in clinical vs. non-clinical populations.

**Methods:**

We conducted a large-scale bibliometric trend analysis of technology-supported PA interventions indexed in Scopus and SportDiscus, covering records published from 1953 to 2025. Using an active-learning screening workflow with title/abstract screening, sensitivity checks and consensus adjudication, we identified 2,981 eligible studies published between 1988 and 2025 across 757 journals and 63 countries. Studies were coded by population (clinical vs. non-clinical), platform cluster, and author-reported PA outcome direction abstracted from the publication record.

**Results:**

Publications increased markedly after 2008, with smartphone/mHealth and wearable sensors becoming the dominant platform clusters in the 2010s and early 2020s. In the smoothed overall trajectories, publication activity reached its highest level around 2022, followed by a contraction concentrated mainly in mature clinical platform clusters. Non-clinical studies generally adopted newer platforms earlier, whereas clinical studies showed a recurrent lag before converging for more established, accessible technologies. A distinct population-level reversal was visible in mature platforms: non-clinical studies led early smartphone, wearable and web-based uptake, clinical studies became more prominent around the COVID-19 period, and non-clinical studies again predominated by 2025 for smartphone/mHealth, wearable sensors and web applications. Multi-component designs were common, with the strongest network backbone centred on smartphone, wearable and web-based combinations. Reported PA improvement signals were common across both population strata, but these findings reflect patterns in published study reporting rather than comparative effectiveness.

**Conclusions:**

By providing a platform-centred, cross-population and temporally resolved map of technology-enabled PA interventions, this study identifies mature technology backbones, emerging areas of experimentation, and recurrent translational gaps between clinical and non-clinical contexts. The findings support more theory-informed and implementation-aware intervention design while underscoring that bibliometric prominence should not be equated with real-world efficacy.

## Introduction

Physical inactivity continues to impose a substantial and preventable burden on global health, contributing to elevated risks of chronic disease, cognitive decline and premature mortality (1, 2). Despite decades of evidence demonstrating the protective effects of regular physical activity, inactivity levels have remained persistently high: in 2016, more than one quarter of adults worldwide were insufficiently active (3), and recent global surveillance suggests that nearly one third still fail to meet recommended activity thresholds (2). This entrenched “pandemic of physical inactivity” (4) threatens hard-won public-health gains and underscores the need for scalable behavioral strategies capable of shifting population-level activity patterns (5). 

Over the past two decades, digital technologies have been increasingly investigated as a response to this challenge. From early web-based programs and computer-tailored interventions in the 2000s to subsequent waves of mobile health (mHealth) platforms, wearable activity sensors, social-media environments and, more recently, AI-enabled conversational agents, technological innovation has progressively expanded the means through which physical activity (PA) can be supported (6, 7). Parallel to this technical evolution, population uptake has accelerated: substantial proportions of adults now routinely use health apps and wearable devices to monitor steps, heart rate or activity patterns (8, 9). Evidence from multiple reviews demonstrates that such tools can produce modest but meaningful increases in PA across a variety of populations, including those traditionally less active, through mechanisms such as self-monitoring, feedback, reinforcement and personalized advice (10–16). These trends highlight the importance of understanding how digital PA platforms have emerged, diversified and been adopted as technologies have matured.

Digital PA interventions have closely followed the broader trajectory of digital innovation: early SMS- and web-based programs with computer-tailored content in the 2000s; smartphone applications in the 2010s, frequently paired with wearable sensors; the rise of social-media communities enabling peer support and fitness challenges; and, most recently, AI-driven conversational agents providing continuous, personalized support via natural-language dialogue (17–26). These successive waves of technological diffusion have shaped how, where and by whom PA interventions can be delivered. At the same time, digital PA interventions can be understood not only as technology platforms but also as vehicles for behaviour change. Platforms such as apps, wearables, telehealth and social media differ in the ways they support self-monitoring, feedback, prompts, social opportunity and personalised guidance. This behavioural lens is important because the practical value of a platform depends not simply on novelty, but on how well it operationalises mechanisms that help people initiate and maintain PA.

Despite their promise, digital PA platforms have not been adopted uniformly across settings (27). A consistent divergence exists between non-clinical contexts, such as public health and consumer wellness, and clinical environments, where implementation has been slower and more constrained (28). In community settings, commercially driven technologies including fitness apps, smartwatches, and online exercise communities have proliferated, with many individuals using them for self-guided fitness and lifestyle management (29). By contrast, clinical uptake remains more constrained: although pilot studies show that tele-exercise programs, remote monitoring wearables, and other digital tools can be feasible and beneficial in areas such as cardiac rehabilitation or diabetes care, sustained integration into routine practice is limited (28). Barriers such as workflow misalignment, data-accuracy concerns, and variable digital literacy among patients and providers further inhibit widespread clinical adoption (30, 31). As a result, technologies that diffuse rapidly in the general population often achieve only niche use in healthcare settings without structured implementation efforts (32). Understanding these contextual determinants is essential, as public-health and clinical stakeholders likely require different strategies to leverage digital PA platforms effectively—for example, broad engagement initiatives in community settings vs. tailored approaches for older or high-risk patients in clinical care. Given the wide variety of digital PA interventions, a structured approach is needed to synthesize research findings. One useful strategy is to organize the evidence by platform clusters, grouping interventions based on the technology modality, such as web-based programs, smartphone apps, wearable devices, telehealth systems or conversational agents (7). This platform-centric organization allows a clearer comparison of how each category has been adopted and combined across settings.

Previous reviews have often narrowed their focus to a single technology type, for instance concentrating exclusively on mobile apps (33) or wearable trackers (34). Others have pooled several modalities under broad eHealth or mHealth labels (12), which makes it difficult to disentangle whether observed patterns are driven by specific platforms, combinations of platforms, or digital interventions in general.

While numerous studies have evaluated digital interventions for physical activity, no comprehensive analysis to date has mapped how distinct platform types are adopted over time across clinical and non-clinical populations while also examining where translational gaps persist. The present paper therefore aims to: (1) describe long-term trends in the use of major digital platform types in PA interventions; (2) contrast their adoption and patterns of author-reported PA improvement in clinical vs. non-clinical populations; and (3) characterise technology–population pairings to identify under-explored areas and future priorities.

## Methods

### Search strategy and eligibility criteria

We conducted a large-scale bibliometric trend analysis of technology-supported physical activity (PA) interventions. To maximise transparency in the search and study-selection process, reporting was informed by PRISMA logic, including a flow diagram summarising identification, screening and inclusion, while the overall reporting approach was also aligned with the preliminary BIBLIO guidance for bibliometric reviews (35). Bibliometric data were retrieved from Scopus (Elsevier) and SportDiscus (EBSCO), selected because together they provide broad multidisciplinary coverage while retaining strong representation of sport, exercise and PA journals. This combination was considered appropriate for a trend-mapping exercise spanning consumer, public-health and clinical literatures, although it was not intended to function as an exhaustive effectiveness review across all biomedical databases (Table 1).

**Table 1:** Eligibility domains and operational definitions used to determine study inclusion and exclusion in the bibliometric review of technology-supported physical activity interventions.

Eligibility domain	Operational definition used in this review
Population	Any population (clinical, non-clinical or mixed) where a digital or device-based intervention aimed to promote, support or increase PA.
Study type	Original empirical evaluations, including randomised and non-randomised trials, pre-post studies, feasibility studies, observational intervention studies, and qualitative/mixed-method evaluations linked to an implemented PA intervention.
Technology focus	Interventions had to use a digital or device-based technology as an active component of PA support rather than measurement only.
Key exclusions	Measurement-only studies, validation/methodological papers, protocols, systematic reviews/meta-analyses, performance-enhancement applications, and qualitative studies focused only on technology development or perceptions without evaluation of an implemented intervention.

### Study selection, screening and data extraction

Searches were conducted in two waves. The first search identified records from Scopus (*n* = 24,596) and SportDiscus (*n* = 7,641), exported on 13 March 2024 and yielding 32,237 references. Duplicates (*n* = 3,280) were removed using Covidence's de-duplication tool and verified manually, leaving 28,957 unique records for screening. A supplementary search covering publications through December 2025 identified 6,226 additional records (6,170 after de-duplication), from which 396 further eligible studies were added after screening and coding. The combined analytic dataset therefore comprised 2,981 eligible studies. In keeping with the bibliometric aims and the size of the corpus, eligibility was assessed at title/abstract level only. This decision enabled consistent screening of the full historical corpus and reflected the primary objective of mapping publication patterns, platform usage and population pairings rather than extracting full-text effect estimates. Screening was conducted by three trained reviewers using ASReview (version 1.0), an open-source machine-learning tool that applies an active-learning algorithm to prioritise records based on predicted relevance. The model was initially trained on 129 known relevant seed articles and a smaller set of clearly irrelevant records. We followed the four-phase SAFE procedure to define transparent stopping rules and safeguards: (1) screening a random 1% sample (*n* = 290) to estimate the proportion of relevant records and refine stopping heuristics; (2) active-learning screening, where reviewers screened 3,994 prioritised records until the stopping criteria were met; (3) applying an alternative model to previously unlabelled records (*n* = 410), yielding 53 further relevant studies; and (4) re-ranking previously excluded records with a simplified model, followed by independent re-screening of 570 records, which identified 226 additional relevant studies. Disagreements or uncertainties were resolved by discussion within the reviewer team, with a senior author adjudicating when consensus could not be reached.

This corpus forms the basis for all subsequent analyses in the present study. In the following sections, we describe (i) the classification of technology platforms, (ii) population cohort categorisation, (iii) temporal trend analyses and trend projections, (iv) technology milestone annotation, and (v) network analyses of technologies and populations.

### Technology platform classification

We manually categorized the intervention technologies from each included study into eight analytically tractable platform clusters: Chatbot/AI, Social Media, Video, Telecommunication/Telehealth, Virtual Reality/Exergaming, Web Applications, Wearable Sensors, and Smartphone/mHealth. These clusters intentionally combine device platforms, delivery modalities and interaction formats because the historical PA literature uses these elements interchangeably when describing interventions; the aim was therefore to harmonize heterogeneous terminology into reproducible, interpretable groupings rather than to claim conceptual equivalence between categories. Each study could include multiple technologies. A manual harmonization and mapping procedure was used to assign every technology mention to one or more clusters (see Supplementary Material S2). Keyword rules included common terms and synonyms. Where a descriptor spanned multiple clusters, a predefined assignment precedence was applied to ensure a reproducible primary label for trend analyses, while co-occurring technologies were retained for the network analysis. Within each study, duplicate mappings were removed so that each cluster was counted only once per record.

### Population cohort categorization

Each included study was further classified by population type to enable stratified analysis of technology trends in clinical vs. non-clinical cohorts. We defined “clinical” studies as those targeting participants with a specific diagnosed health condition or disease (e.g., diabetes, cardiovascular disease, cancer survivorship, obesity or other medical diagnoses), and “non-clinical” studies as those involving generally healthy populations or community samples without a stated diagnosis. This categorization reflects participant diagnostic status rather than the degree of implementation within formal health-care delivery systems; several studies classified as clinical were still prevention-oriented or research-led rather than embedded in routine care. The original dataset contained over 150 distinct health conditions across the clinical studies, and each record was labelled on the basis of the participant description reported in the publication.

### Temporal trend analysis

Using the categorized data, we analyzed long-term temporal trends in the use of each technology platform cluster, overall and by population cohort. Each included study contributed one count to each relevant platform cluster in the year of its publication. Thus, for each calendar year, we computed the number of published PA intervention studies that utilized technologies in each cluster. We performed this aggregation separately for clinical vs. non-clinical studies, yielding annual counts by cluster and cohort. The time span of the analysis was determined by the publication years of included studies. To visualize and compare longitudinal trajectories, we generated trend lines for each technology cluster within each cohort. Because the annual counts in early years were very low (and zero for clusters that had not yet emerged), and year-to-year fluctuations can be noisy, we applied a 3-year centered moving average to smooth the trend lines. Specifically, each year's value was replaced by the average of that year, the year prior, and the year after (at the endpoints of the series, a 2-year average was used). This smoothing dampens short-term variability and highlights underlying trends over multi-year periods. The smoothed trends were then examined to identify periods of introduction, growth, and possible saturation for each platform cluster. We also calculated the proportional uptake of each cluster over time (e.g., the percentage of all intervention studies in a given year that used a given technology) to complement raw counts, allowing us to gauge the relative prominence of different technology types in the literature at any point. These analyses enabled a descriptive mapping of how digital platform utilization in PA interventions has evolved annually and how patterns differ between clinical and non-clinical contexts. All analyses were performed using Python and R, with results cross-validated by two analysts to ensure accuracy in coding and counting.

### Trend projection

To explore potential short-term trajectories, annual publication counts for each technology cluster were summarised for calendar years 2016–2025 and analysed using simple linear regression as a heuristic foresight exercise. Calendar year was treated as a continuous predictor and annual publication count as the outcome. Separate models were estimated for each cluster and, where relevant, for each population subgroup. The estimated slope for 2016–2025 was extrapolated to 2026–2030 to produce illustrative scenario projections. These projections are not forecasts of actual adoption, but simple extensions of recent publication trajectories intended to support discussion of possible near-term directions under recent trend assumptions.

These scenario projections were performed separately for clinical and non-clinical study counts to estimate whether the gap between cohorts might narrow or widen under recent trend assumptions. They do not account for external limiting factors, nonlinear saturation, market shifts, regulatory changes, or publication lag. For the Chatbot/AI cluster, which had a very low baseline and a sharp recent increase, we implemented a constrained ramp scenario. The linear model was fitted to observed data for 2016–2025, while extrapolated values for 2026–2030 were capped by a conservative maximum year-on-year increment. The cap affected only extrapolated values and did not alter empirical study counts. All projections for 2026–2030 are therefore presented as illustrative scenarios rather than precise forecasts, and no statistical inference was performed on projected values.

### Technology milestone annotation

To contextualize the trends, we identified and annotated major technological development milestones relevant to each platform cluster. We conducted a brief historical review of digital technology innovations that have impacted PA intervention delivery, drawing on commercial release records, technical documentation, and prior literature. Key milestones were selected for their influence on mainstream availability or adoption of a given platform. These events were added as vertical reference lines on the trend figures (with annotations) to indicate when a technology breakthrough or product launch occurred relative to publication trends. Notable examples include the advent of smartphones, wearable, and AI technologies: the first SMS text message was sent in 1992, marking the beginning of mobile phone text communication (Telecommunication/Telehealth); the launch of Facebook in 2004 popularized online social networking (Social Media); the founding of YouTube in 2005 enabled widespread sharing of online video content (Video); the release of Apple's iPhone in 2007 revolutionized the smartphone app ecosystem and mobile health tracking (Smartphone/mHealth); the introduction of the Fitbit tracker in 2009 brought wearable activity monitors to the consumer market (Wearable Sensors); the Oculus Rift's consumer debut in 2016 signaled the arrival of affordable, high-quality virtual reality for health and exercise applications (Virtual Reality/Exergaming); and the public release of OpenAI's ChatGPT in 2022 demonstrated a leap forward in AI chatbot capabilities, catalyzing interest in conversational AI for health interventions (36) (Chatbot/AI). These milestones (and others for completeness) were annotated on the graphs with dotted vertical lines at the corresponding years. Each selected event was documented with a source reference—for example, contemporaneous reports or academic reviews of the technology—to provide evidence of its timing and significance. By overlaying these historical markers on the trend lines, we aimed to illustrate how surges or inflections in the adoption curves may align with the introduction of enabling technologies. All milestone annotations were finalized by consensus of the research team. Through these annotated timelines, the Methods provide a historically informed portrayal of when and how various digital platforms entered the scene of physical activity promotion, supporting a nuanced interpretation of the observed trends. This approach thereby enables a comprehensive understanding of the interplay between technological advancements and their subsequent integration into mHealth interventions for physical activity.

### Network analysis

We grouped each study's reported technologies into the eight platform clusters described above. Per study, duplicate clusters were collapsed. Clusters were mapped as nodes on a circular layout with node size proportional to cluster frequency. When a study used multiple clusters, undirected links were drawn between the relevant nodes, with link width reflecting total co-occurrence across studies. Two population nodes (Clinical and Non-clinical) were added. For each cluster–population pair, we calculated the proportion of studies reporting a positive PA-related outcome direction, excluding records coded as “not clear”. These outer links therefore reflect author-reported outcome patterns abstracted from the publication record, not comparative effectiveness, causal impact or risk-of-bias–adjusted evidence. Because these outcome-direction codes were derived from titles and abstracts, they should be interpreted as a high-level bibliometric signal only.

## Results

A total of 2,981 studies met the inclusion criteria, spanning publications from 1988 through 2025. Studies originated from 63 countries and were published across 757 different journals, underscoring the breadth and disciplinary diversity of this literature. The study-selection workflow is summarised in Supplementary Figure S1. Results are organised below according to the three study aims.

### Aim 1: long-term trends in major digital platform types

**Figure 1:**
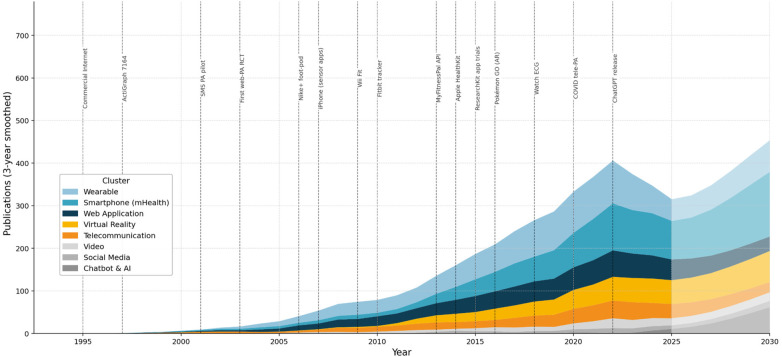
Technology clusters in physical activity-related digital health publications (1995–2030). Observed publication counts are shown through 2025; post-2025 values represent exploratory scenario projections based on recent trend windows rather than forecasts.

Overall, the annual number of technology-supported physical activity intervention publications increased markedly over time (Figure 1). Only three papers were identified in the 1988–1997 period, rising to 172 papers in 1998–2007. In the three-year smoothed stacked trajectory shown in Figure 1, overall platform activity reached its highest level around 2022 and then contracted through 2025, while remaining substantially above pre-2018 levels. An inflection point occurred in the late 2000s, after which publication counts expanded rapidly. Between 2008 and 2017, the field saw approximately 1,000 publications, reflecting a roughly six-fold increase from the previous decade. This steep upward trajectory continued into the most recent years: 2018–2025 alone yielded 1,807 publications, exceeding the output of the entire prior decade in just eight years.

The cumulative trend illustrates an exponential rise in research activity using digital platforms for physical activity promotion, especially post-2010. Notably, different technology platform clusters rose to prominence at different times, leading to shifts in the composition of interventions over the years. Early growth in the 1990s and early 2000s was driven primarily by a few foundational technologies. Interventions using basic wearable sensors (especially first-generation devices like pedometers) (37), web-based applications (desktop computer or internet programs), and legacy telecommunication methods (telephone counseling and simple text messaging) constituted the majority of early studies. For instance, of the 175 total publications through 2007, approximately 79% employed one of these early platforms. The mid- to late-2000s marked a turning point: following the introduction of consumer smartphones and advanced fitness trackers, mobile application–based interventions and second-generation wearables experienced a rapid surge. Publications involving smartphone (mHealth) platforms and modern wearable trackers began to overtake those based on earlier technologies by the mid-2010s. As a result, the overall growth from 2008 to 2017 was largely attributable to the proliferation of these two platform clusters. During this phase, previously dominant approaches showed signs of plateauing: for example, web application–based studies grew more slowly and leveled off, and publications relying on traditional telecommunication or video-based content reached a peak and then declined in relative share. In Figure 1, the steepest visible transition occurs after the annotated smartphone and consumer wearable milestones, when the cumulative contribution of smartphone/mHealth and wearable platforms begins to exceed the earlier web-, video- and telecommunication-centred layers.

In the most recent period (late 2010s through 2025), the data indicate both continuity and further transition in platform use. Smartphone and wearable platforms remained the most frequently used technologies by the 2020s, together comprising the largest portion of new interventions. Traditional web-based interventions showed a slower trend in the 2020s, while earlier video- and mass-media-oriented approaches contributed a smaller share of the literature. Meanwhile, several emerging platform clusters appeared in the late 2010s and early 2020s. Social media and virtual reality contributed smaller but persistent study volumes, and Chatbot/AI platforms rose from near-zero levels to a visible, though still modest, segment by 2025. After the 2022 ChatGPT milestone, the steepest increase was concentrated in Chatbot/AI, whereas several established platform curves flattened or declined. Virtual reality and social media did not reach the volume of the dominant smartphone and wearable clusters, but their curves were less visibly affected by the post-2022 contraction than several mature platforms.

### Aim 2: adoption patterns and author-reported PA outcome direction in clinical vs. non-clinical populations

**Figure 2:**
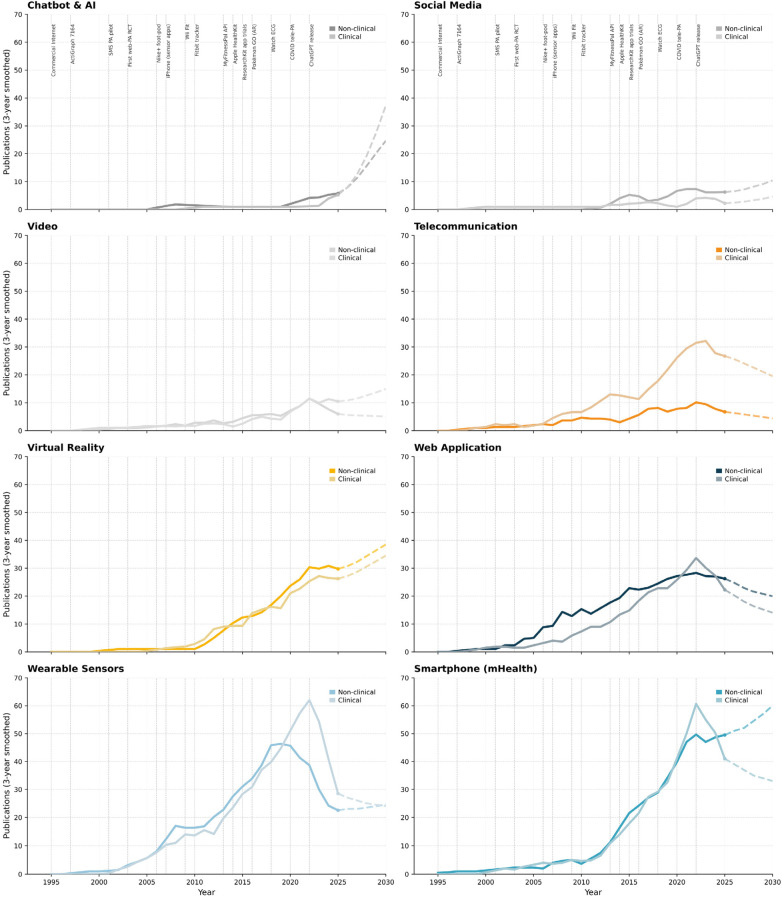
Platform clusters in physical activity-related digital health publications (1995–2030), stratified by population. Shading: non-clinical = base hue; clinical = desaturated hue. Observed trends are separated from post-2025 exploratory scenario extensions to reduce over-interpretation of projected values.

Stratifying the publication trends by target population reveals differences in both timing and magnitude of technology adoption between clinical and non-clinical settings (Figure 2). Non-clinical studies were generally quicker to incorporate newer digital platforms, particularly social media, virtual reality and early AI-linked approaches. For more established technologies, especially smartphones, web applications and wearable sensors, clinical publications appeared later but increased sufficiently during the 2010s and early 2020s to narrow or temporarily reverse the initial gap.

A temporal reversal is visible across several mature platforms. In 2018–2019, non-clinical publication counts were higher than clinical counts for smartphone/mHealth and wearable sensors. During the COVID-19 period and its immediate aftermath, clinical counts matched or exceeded non-clinical counts in several mature clusters, most clearly for telecommunication/telehealth, wearable sensors and smartphone/mHealth. By 2025, the pattern had shifted again: non-clinical counts were higher than clinical counts for smartphone/mHealth, wearable sensors and web applications.

The post-2022 decline was more pronounced in clinical than in non-clinical studies for several established clusters. This was particularly visible for wearables, smartphone/mHealth and web applications, where clinical publication counts fell more sharply after their 2021–2023 peaks. Telecommunication/telehealth showed a distinctive clinical surge around the COVID-19 milestone, followed by a contraction after 2022. In contrast, Chatbot/AI remained small but increased in the final years of observation, with clinical studies only becoming clearly visible at the end of the series. Virtual reality and social media remained smaller than the dominant smartphone and wearable clusters, but their trajectories were comparatively less tied to the post-COVID decline in mature clinical platforms. The population ordering therefore changed over time: early non-clinical predominance was followed by a temporary clinical surge around COVID-19 and then a return to non-clinical predominance by 2025 for several mature platforms.

### Aim 3: technology-population pairings, under-explored areas and future priorities

**Figure 3:**
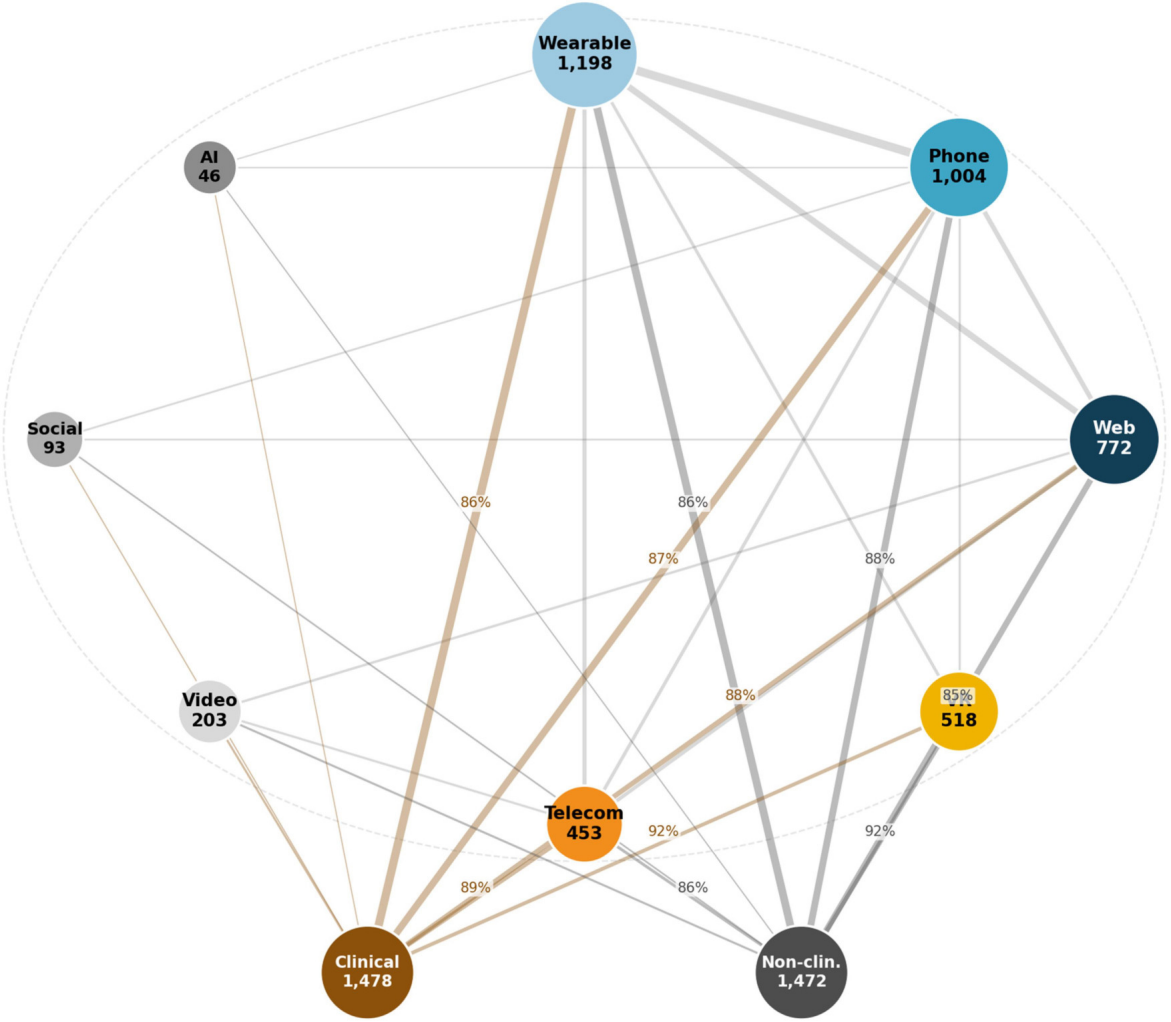
Network plot of technology clusters used in digital physical-activity interventions across all included studies. Inner ring shows cluster co-occurrence within interventions; outer ring shows connections to Clinical and Non-clinical population nodes based on author-reported PA outcome direction abstracted from the publication record. Node size reflects cluster frequency in the final dataset; percentage labels on outer links are descriptive outcome-direction proportions among records with clear coding.

As a next step, network analysis (Figure 3) was used to map how technology clusters co-occur within interventions and how they link to clinical vs. non-clinical populations. Across included studies, clinical and non-clinical populations were almost equally represented (clinical *n* = 1,478; non-clinical *n* = 1,472; mixed *n* = 31). Wearable sensors, smartphone/mHealth and web applications formed the core of the network, with the strongest co-occurrence links observed among smartphone, wearable and web-based platforms. Telecommunication/telehealth was also strongly connected to wearable and web-based clusters, indicating that remote delivery was frequently embedded in multi-component designs rather than appearing only as a stand-alone platform. In contrast, social media and Chatbot/AI occupied smaller and more peripheral positions. Outer links showed that the central platform clusters connected strongly to both clinical and non-clinical populations, whereas AI and social-media links remained sparse and should be interpreted cautiously.

## Discussion

This bibliometric study aimed to map how the technological architecture of PA promotion research has evolved over time. We identified 2,981 relevant publications from 1988 to 2025, reflecting a major expansion of the evidence base. These studies were published across 757 journals and represented research from 63 countries, indicating broad international interest in technology-supported PA interventions. Taken together, the findings suggest a field that has expanded rapidly, become increasingly centred on smartphone and wearable ecosystems, and only recently begun to explore newer modalities such as immersive systems and AI. The discussion below focuses on what these patterns imply for mechanism, translation and future research priorities rather than restating the full descriptive results.

A clear temporal pattern emerged in the types of technology employed. Early on (circa 1988–2007), the number of technology-based PA studies was relatively small (175 studies in that entire period) and was dominated by what might be considered first-generation digital tools. In that era, simple pedometers and other early wearables, mass media campaigns or messaging, and desktop computer-based interventions were the primary platforms used. These simpler technologies accounted for roughly 79% of all studies in 1988–2007. Following this foundational period, there was rapid growth during the late 2000s and 2010s, driven largely by second-generation wearables and smartphones with dedicated PA apps. By the late 2010s and early 2020s, attention had increasingly shifted to more sophisticated, data-rich platforms. AI-enhanced interventions such as chatbots, virtual coaches and machine-learning-guided recommendations began to appear, but still represented a small fraction of the overall literature. The field therefore appears to have developed through successive waves: early web and messaging infrastructure, mobile and sensor consolidation, and a more recent phase of AI- and immersive-technology experimentation. Importantly, the post-2022 contraction in the smoothed mature-platform curves does not suggest that digital PA research has declined as a whole; rather, it suggests a redistribution of publication activity across platform labels and settings.

The network analysis reveals a mature backbone of wearable–smartphone–web platforms that are both common and frequently combined, while newer modalities such as VR and Chatbot/AI remain less prevalent. Where sample sizes are sufficient, author-reported positive PA outcome proportions are high in both clinical and non-clinical groups, with only modest differences between them. This should not be read as evidence that any platform is definitively more effective; rather, it shows which technologies have been repeatedly studied and positively reported. The centrality of multi-component designs, especially smartphone–wearable combinations, also indicates that the field has moved beyond single-platform interventions. Future studies should therefore test whether common pairings add value beyond simpler designs, rather than assuming that technological layering automatically improves outcomes.

A related point is that apparent decline in a platform category does not necessarily mean disappearance from practice. Web applications and telecommunication may increasingly function as infrastructural layers within mobile, wearable or AI-enabled systems rather than as stand-alone intervention labels. This is especially important when interpreting the post-2022 pattern: a decline in web- or mHealth-labelled clinical studies may partly reflect platform convergence and changing terminology, not simply reduced interest in digital delivery. In other words, mature platforms may be becoming less visible as independent categories precisely because they are being absorbed into more integrated intervention architectures.

### Comparison with previous research

Our findings align with and expand upon observations from prior conceptual and review papers (7). We document the modest beginnings and subsequent plateau of earlier tools like desktop and SMS-based interventions, the rise of mobile app and wearable interventions roughly a decade ago, and the nascent uptake of AI methods in recent years (38). This temporal pattern also mirrors broader consumer technology trends—for instance, the advent of the iPhone and app ecosystems in 2007–2008 spurred many app-based PA programs, while more recently the advances in AI have prompted researchers to experiment with chatbots for exercise coaching. In essence, PA intervention research has kept in step with technological innovation cycles, a synergy noted by other authors as well. Calabuig-Moreno et al. (39), for example, performed a bibliometric analysis focusing on virtual and augmented reality applications in physical education, reflecting the growing interest in immersive technologies for activity promotion (39). While their scope was narrower, centered on VR/AR in educational settings, our results indicate that such immersive “media” technologies (part of our media/TV-based category) still represent a relatively small niche (on the order of a few hundred studies) in the overall landscape. This suggests that despite hype around newer technologies, traditional platforms like mobile apps remain more widely studied at the population level.

In line with our results, recent systematic reviews and scoping reviews of digital health interventions for PA have found that mobile applications and wearable trackers dominate the field (12, 40). For instance, a scoping review of 40 reviews by De Santis et al. (41) noted that most primary studies in those reviews targeted adult populations and “focused on mobile apps or wearables for physical activity promotion” (41). This independent observation reinforces our finding that smartphones and wearable devices became the centerpiece of PA promotion efforts in the 2010s. A likely explanation is the capability of these tools to provide real-time feedback and convenient self-monitoring, which are known to enhance engagement in behavior change interventions (41). Indeed, evidence is emerging that such technologies can yield positive short-term outcomes. Woessner et al. (42) reviewed the impact of modern tech on inactivity and reported that providing individuals with activity trackers tends to increase their daily step counts and even yield modest health benefits (e.g., improved mobility or slight reductions in BMI) in the short term (42, 43). Gamified and app-based interventions have also shown success in boosting enjoyment and motivation for exercise in initial studies (44, 45). These findings underscore why wearables and apps have been so prevalent—they are not only widely accessible but can be effective at least in initiating behavior change. However, the same reviews caution that the long-term effectiveness of these technologies remains uncertain, as many interventions are of short duration and user adherence often wanes over time (46). This highlights a critical need for future research to explore strategies for sustaining engagement with digital health interventions over extended periods, particularly through features that adapt to evolving user needs and preferences (47, 48).

Our bibliometric analysis captures the rapid proliferation of research on wearables and apps, but, by design, it cannot determine whether these platforms sustain PA beyond the initial novelty period or outperform alternatives under comparable conditions. Interpreted through the Behaviour Change Wheel and COM-B model (49), however, the dominance of these platforms is not surprising: many are particularly well suited to self-monitoring, prompts, feedback and action planning, and therefore may be especially visible in the literature because they operationalize core behaviour change functions in a scalable digital format. Nonetheless, previous work suggests that maintaining behavior change remains difficult, and emerging AI- and machine-learning–driven approaches to hyper-personalized, just-in-time adaptive interventions may offer one way to address this challenge (50). An additional consideration is which technologies are used at what point in time in PA studies. For example, exergame research in clinical settings often relies on outdated systems (e.g., trials published in 2018 still using the Nintendo Wii, even though the Nintendo Switch was already available). This suggests that clinical contexts may systematically lag behind consumer markets in adopting newer technologies, which in turn shapes which tools are evaluated and thus the publication patterns captured in our bibliometric analysis. Future studies should therefore focus not only on developing new digital tools, but on designing “exit by design” interventions that build people's skills, confidence and routines so that physical activity can be sustained with progressively less reliance on technology. In this view, digital platforms act as temporary scaffolds that can be stepped down, exited, and later re-engaged or intensified when needed, rather than as perpetual “prescriptions” for activity—an issue also raised by Woessner et al. and others. Moreover, the efficacy of persuasive technologies in promoting physical activity has been a significant area of focus, predominantly leveraging mobile applications and wearable devices (51).

Another notable finding is the substantial number of technology-based PA interventions targeting clinical populations (1,478 studies, covering around 150 distinct health conditions). This reflects an important intersection of digital health with chronic disease management (52). At the same time, it should not be read as evidence that these interventions are already well integrated into routine care; rather, it reflects how often diagnosed populations appear in the research record. Previous research supports the idea that digital PA interventions can be valuable in clinical contexts: for example, an overview of systematic reviews by Kardan et al. (1) found that interventions such as wearable activity trackers and telehealth platforms can increase physical activity among patients with major non-communicable diseases (1). The effectiveness evidence, however, came with an important caveat: Kardan et al. noted that the quality of many digital intervention studies was low and should be interpreted cautiously. This aligns with our own observation that research volume does not automatically equate to robust evidence for real-world impact. From an implementation perspective, the recurrent clinical lag is also consistent with the NASSS framework (53), which suggests that technology uptake in health care is shaped not only by the tool itself but also by clinical workflows, organisational readiness, value proposition and the wider institutional context.

The population-stratified trends add a further nuance: the newest period is not simply a continuation of clinical catch-up. For mature platforms, clinical publication counts rose strongly around the COVID-19 period and then declined more sharply than non-clinical counts after 2022, particularly for wearable sensors, web applications and smartphone/mHealth. This temporal pattern coincides with, but cannot be attributed causally to, the release of ChatGPT and the broader shift toward AI-enabled digital health. One plausible explanation is that clinical digital PA research is more dependent on service structures, funding cycles, regulatory expectations and implementation constraints than non-clinical research. Another is that some innovation previously described as app-, web- or wearable-based is beginning to be reframed as AI-enhanced or adaptive support. A third possibility is that emergency-driven clinical digitisation during COVID-19 temporarily accelerated remote and sensor-supported intervention research, followed by partial normalisation once acute service disruption eased. The finding does not make the clinical domain less relevant; instead, it suggests that clinical uptake is more sensitive to external shocks and changes in the surrounding technology ecosystem.

Finally, our analysis captures only the early published development of AI-driven approaches to PA promotion. Chatbot/AI appears promising but remains an early-stage and still thinly evidenced area of the literature (54). Only 46 studies were classified in the Chatbot/AI cluster, with a sharp uptick in recent years. Because there is typically a lag between the availability of new AI technologies, securing funding, conducting trials and publishing results, the current bibliometric snapshot almost certainly underestimates AI-based innovation currently underway. Other scholars have begun to discuss what this could entail, including digital humans, AI-powered virtual coaches and conversational agents that may support tailoring, companionship and feedback in PA programmes (55, 56). Our data show that the research community has started to explore this frontier, but the evidence remains exploratory. Rather than implying an imminent dominant trend, AI-related findings should be read as signals of experimentation that require rigorous evaluation, transparent reporting and implementation testing before stronger translational claims are warranted (57). The key question is therefore not whether AI will replace established platforms, but whether it can add adaptive decision support to intervention components already shown to be feasible, such as mobile delivery, wearables, telehealth and human coaching.

### Limitations and implications

While this analysis provides a comprehensive overview of technology use in PA promotion research, several limitations must be acknowledged. First, our data were sourced exclusively from Scopus and SportDiscus. Although this pairing offered broad multidisciplinary and sport-specific coverage, relevant studies indexed only in other databases may have been missed, so absolute counts should be interpreted as conservative estimates of the total literature. Second, inclusion was based on title/abstract screening, which may have missed studies that did not clearly report the technological intervention in the abstract. We mitigated this risk through active-learning safeguards, manual checks and consensus adjudication, but some omissions remain possible. Third, we did not assess study quality or comparative effectiveness; all included papers contribute equally to the bibliometric map regardless of design strength, follow-up duration or risk of bias. The bibliometric approach is suited to mapping trends and volumes of research, but it cannot tell us which technologies are most effective in increasing physical activity or improving health. Readers should therefore avoid interpreting platform prominence as proof of efficacy. Publication bias may also distort the visibility of platforms that appear especially promising in the published record, while lag between consumer-market innovation and academic evaluation means that some technologies are already outdated when results become available. Both dynamics are particularly relevant in fast-moving areas such as wearables, immersive technologies and AI.

We also acknowledge classification and data limitations. We grouped technologies into eight primary categories for analytical clarity, but in reality these categories sometimes overlap. Many interventions used multiple technologies together (58% used two or more), yet trend analyses still require simplified labels. This means nuances can be lost; for example, a combined intervention using a website, wearable and app may not be fully represented by a single dominant platform. Moreover, the definition of certain categories, such as what qualifies as social media or AI, requires judgement and may shift as technologies converge. To reduce this loss of nuance, we retained co-occurring technologies in the network analysis even when a single primary label was required for temporal trend plots. Finally, we focused on published studies of interventions or evaluations. Real-world commercial and community use of fitness technologies is not captured in this academic literature, which highlights a continuing gap between research trends and everyday practice.

The findings carry several important implications for research. Understanding the trajectory of technology use in PA interventions can help identify under-explored areas and avoid redundancy. For example, the relative paucity of AI-based intervention studies to date suggests an opportunity for researchers to rigorously test and refine such approaches, beyond the 46 studies identified here. However, as the field adopts new tools, it is important to address persisting questions about long-term effectiveness, engagement and implementation. A behaviour change lens may help here: the COM-B model suggests that future interventions should be explicit about which combinations of capability, opportunity and motivation they are targeting, and how a given technology platform is expected to deliver those functions in practice (49). Another research implication is the need for greater consistency in how digital interventions are evaluated and reported (58). Standardising outcome measures, engagement metrics and terminology would strengthen future synthesis and help distinguish genuine platform effects from reporting artefacts (59). Where sufficient outcome data are available, advanced meta-analytic approaches may further support the synthesis of heterogeneous or reconstructed patient-level outcomes (60). The network results also point to a methodological priority: common combinations such as smartphone-plus-wearable designs should be tested against simpler alternatives so that the field can identify when technological layering adds value and when it adds complexity without clear benefit.

For practitioners and public health professionals, our results underscore the value of aligning interventions with technologies that are already embedded in users' everyday lives, while avoiding the assumption that popularity alone guarantees impact (61). The rapid increase of mobile- and wearable-based studies in the 2010s reflects the practical appeal of platforms that support self-monitoring, prompts, feedback and remote tailoring at scale (62). However, the post-2022 clinical contraction suggests that clinical implementation cannot rely on technological availability alone. In clinical contexts, platform choice must also fit care pathways, professional workflows, safety expectations and reimbursement structures. Importantly, the human element should not be neglected: technology is a vehicle for behaviour change, but coaching, social support and personal relevance remain crucial (63, 64). Framed through COM-B, the most promising platforms are not necessarily the newest ones, but those that reliably support motivation, create opportunities for action and reduce capability barriers for the intended user group (49). The NASSS framework (65) further reminds us that successful scale-up depends on fit across the condition, the technology, the adopter system, the organisation and the broader context, not on technical novelty alone (53).

Policy and governance conditions will also shape which platforms remain feasible. This is particularly relevant for social-media-based PA interventions, which may appear attractive because of reach and peer-support potential but are increasingly subject to age-assurance, safeguarding and platform-accountability requirements. Australia's under-16 social-media account restrictions illustrate how quickly the implementation environment for youth-facing digital interventions can change (66). For PA promotion, this does not necessarily argue against social media, but it suggests that future interventions may need to rely more on moderated communities, consent-aware designs, age-appropriate platforms, or integration with schools, health services and community organisations rather than open commercial social networks alone.

## Conclusion

Across nearly four decades of research, technology-enabled PA interventions have shifted from early web-, messaging- and telecommunication-based formats toward smartphone and wearable ecosystems, with newer modalities such as immersive platforms, social media and AI remaining comparatively nascent. Non-clinical settings typically adopted new platforms earlier, whereas clinical populations showed both translational lag and greater sensitivity to external disruption, particularly around COVID-19 and the post-2022 platform transition. These patterns help identify where the literature is mature, where evidence remains thin, and where theory-informed and implementation-aware research is most needed. At the same time, the present study maps publication and reporting patterns rather than comparative effectiveness; future work should therefore pair innovation with stronger evaluation, clearer behavioural logic and greater attention to scale-up in real-world settings. Clinical applications are not becoming irrelevant; rather, the findings suggest that clinical uptake is especially dependent on implementation context, regulatory conditions and the stability of the surrounding delivery infrastructure.
