# Supplementary material for: The past, present and future use of technology-enabled physical activity interventions in clinical and non-clinical populations: a bibliometric trend analysis across four decades
Source: Front Digit Health. 2026 May 29;8:1801405. doi: 10.3389/fdgth.2026.1801405 (PMC13262197; doi:10.3389/fdgth.2026.1801405)
Supplement: Supplementary file 1 [file Supplementaryfile1.pdf]

Supplementary Material 1: Prisma Flow Chart

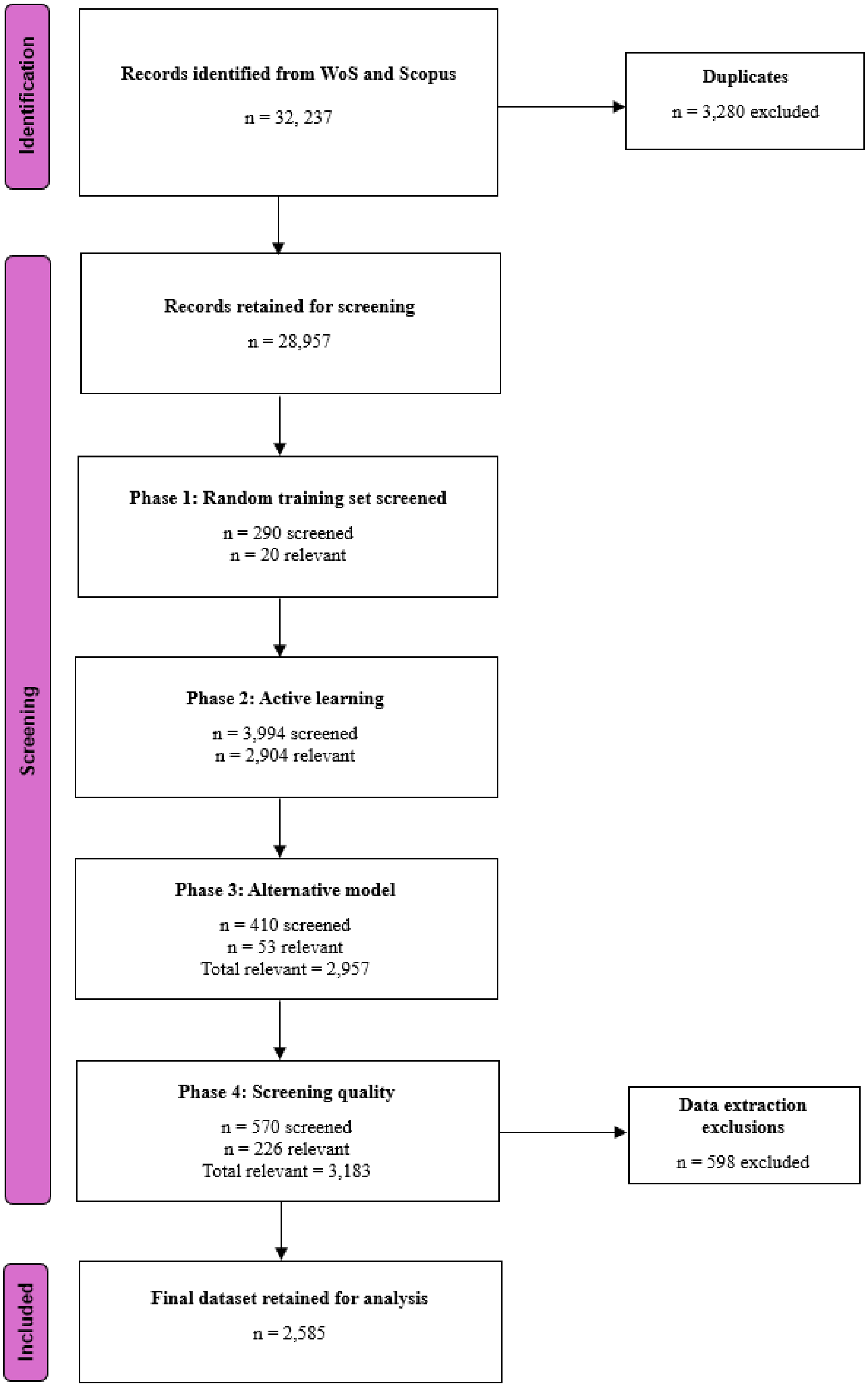

Supplementary material 2: Summary of Clusters

Cluster	Studies in this cluster (n)	Most frequently included technologies
**Chatbot & AI**	46	Digital assistant (n=11), Artificial intelligence (n=10), App (n=7), Chatbot (n=7), Mobile phone (n=6)
**Smartphone (mHealth)**	1,004	Mobile phone (n=602), App (n=460), Text/SMS (n=248), Telehealth (n=98), Pedometer (n=93)
**Social Media**	93	Social media (n=86), Mobile phone (n=19), Text/SMS (n=19), Website (n=19), App (n=16)
**Telecommunication**	453	Telehealth (n=451), Pedometer (n=75), Mobile phone (n=46), Website (n=34), Text/SMS (n=33)
**Video**	203	Video (n=155), Telehealth (n=38), Website (n=35), Mobile phone (n=27), Text/SMS (n=24)
**Virtual Reality**	518	e-game (n=271), Virtual (unspecified VR environment, n=132), Gaming console (n=82), Active gaming (n=73), Augmented reality (n=61)
**Wearable Sensors**	1,198	Pedometer (n=572), Accelerometer (n=223), Mobile phone (n=220), App (n=160), Activity tracking device (n=150)
**Web Application**	772	Website (n=409), Internet (n=154), Email (n=138), Pedometer (n=110), Mobile phone (n=94)
